# BRIDES: A New Fast Algorithm and Software for Characterizing Evolving Similarity Networks Using Breakthroughs, Roadblocks, Impasses, Detours, Equals and Shortcuts

**DOI:** 10.1371/journal.pone.0161474

**Published:** 2016-08-31

**Authors:** Etienne Lord, Margaux Le Cam, Éric Bapteste, Raphaël Méheust, Vladimir Makarenkov, François-Joseph Lapointe

**Affiliations:** 1 Département d'informatique, Université du Québec à Montréal, Montréal, Québec, Canada; 2 Département de sciences biologiques, Université de Montréal, Montréal, Québec, Canada; 3 Sorbonne Universités, UPMC Université Paris 06, Institut de Biologie Paris-Seine, Paris, France; 4 CNRS, UMR7138, Institut de Biologie Paris-Seine, Paris, France; College of Bioinformatics Science and Technology, CHINA

## Abstract

Various types of genome and gene similarity networks along with their characteristics have been increasingly used for retracing different kinds of evolutionary and ecological relationships. Here, we present a new polynomial time algorithm and the corresponding software (BRIDES) to provide characterization of different types of paths existing in evolving (or augmented) similarity networks under the constraint that such paths contain at least one node that was not present in the original network. These different paths are denoted as **B**reakthroughs, **R**oadblocks, **I**mpasses, **D**etours, **E**qual paths, and **S**hortcuts. The analysis of their distribution can allow discriminating among different evolutionary hypotheses concerning genomes or genes at hand. Our approach is based on an original application of the popular shortest path Dijkstra’s and Yen’s algorithms. The C++ and R versions of the BRIDES program are freely available at: https://github.com/etiennelord/BRIDES.

## Introduction

Network structures provide useful representations of interactions between the elements of complex systems [1, 2]. They can, for example, represent relationships between microbial communities in different environments [3] or between proteins in different bacteria and eukaryotes [4]. The abundance of the network elements (i.e. represented by nodes) as well as their interactions (i.e. represented by edges) often vary over time. Comparing evolving networks containing sets of attributes (or annotations) at their nodes is currently becoming central to different fields of biology, including ecology, evolution, cell biology and medicine [1, 5–7]. For example, genome similarity networks, where each node represents a genome and the edge weights correspond to the number of shared gene families between genomes, have been used to identify horizontal gene transfer events [8] and other reticulate phylogenetic relationships [3]. Genome similarity networks are typically constructed at different stringency thresholds (e.g. 50, 60, 70, 90, 99%) [6], or by using constantly increasing datasets, thus producing a range of inclusive networks. Such a strategy allows the detection of ancient evolutionary connections [1] or the verification of ecological distribution of taxa [9]. Furthermore, network analysis can be used with heterogeneous types of biological data, e.g. for linking protein structures to their functions [10,11].

Previous works in this field have focused on the use of conventional graph-theoretic measures describing the evolution of networks, such as the numbers of nearest neighbors or certain network motifs [12,13], or the distribution of shortest paths [9]. In this study, we present a number of novel features, which characterize evolving networks. All of them are based on the presence of additional nodes and edges in the augmented network. These *added nodes* and *edges* can be used to connect the original network nodes through different types of simple paths (i.e. loopless paths or paths that do not visit the same nodes twice) [14,15]. Precisely, we will describe a new polynomial time algorithm for estimating the number of **B**reakthroughs, **R**oadblocks, **I**mpasses, **D**etours, **E**qual paths and **S**hortcuts (BRIDES) in evolving networks. Moreover, we have developed C++ and R functions implementing the new algorithm. We will also present the results of our simulation study comparing the performances of four different versions of our algorithm as well as the application of the most successful version of BRIDES to real genome similarity networks. It is worth noting that, contrary to previous work, we were neither interested in counting the number of colored motifs in networks [16], nor in determining their types [12, 13].

## Materials and Methods

### Description of the BRIDES algorithm

This section describes the problem we address here from a mathematical point of view and presents the most important computational details of our algorithm. The main questions that we try to answer in this paper are the following:

1. Is there a simple path between two given nodes *i* and *j* (i.e. original network nodes in our study) that contains at least one node from a specific set of nodes (i.e. set of added nodes in our study)?
2. If such a simple path exists, is it the shortest path between *i* and *j*?

Since the number of simple paths between two given nodes in a graph can be exponential in the size of the graph, visiting and counting all of them is a problem belonging to #P [14, 17]. On the other hand, the number of simple shortest paths between two nodes of an undirected graph can also be exponential in the number of the graph nodes. Furthermore, the problem of finding a simple shortest path including a set of *must-include* nodes is NP-hard [18, 19]. Therefore, effective heuristic algorithms should be applied to answer questions (a) and (b), especially when large genetic or genomic similarity networks are considered. Vardhan and colleagues [19] proposed a fast heuristic algorithm to compute a simple path that contains a given ordered set of must-include nodes. However, this problem is slightly different from our problem, since our main objective is to find a shortest simple path including *at least one node* from a set of specified nodes. Li and colleagues [20] tried to address the latter problem, presenting a fast heuristic approach based on the principle of optimality of dynamic programming. However, their elegant algorithm can be applied only to graphs with a specific–series/parallel–topology [20].

**Fig 1:**
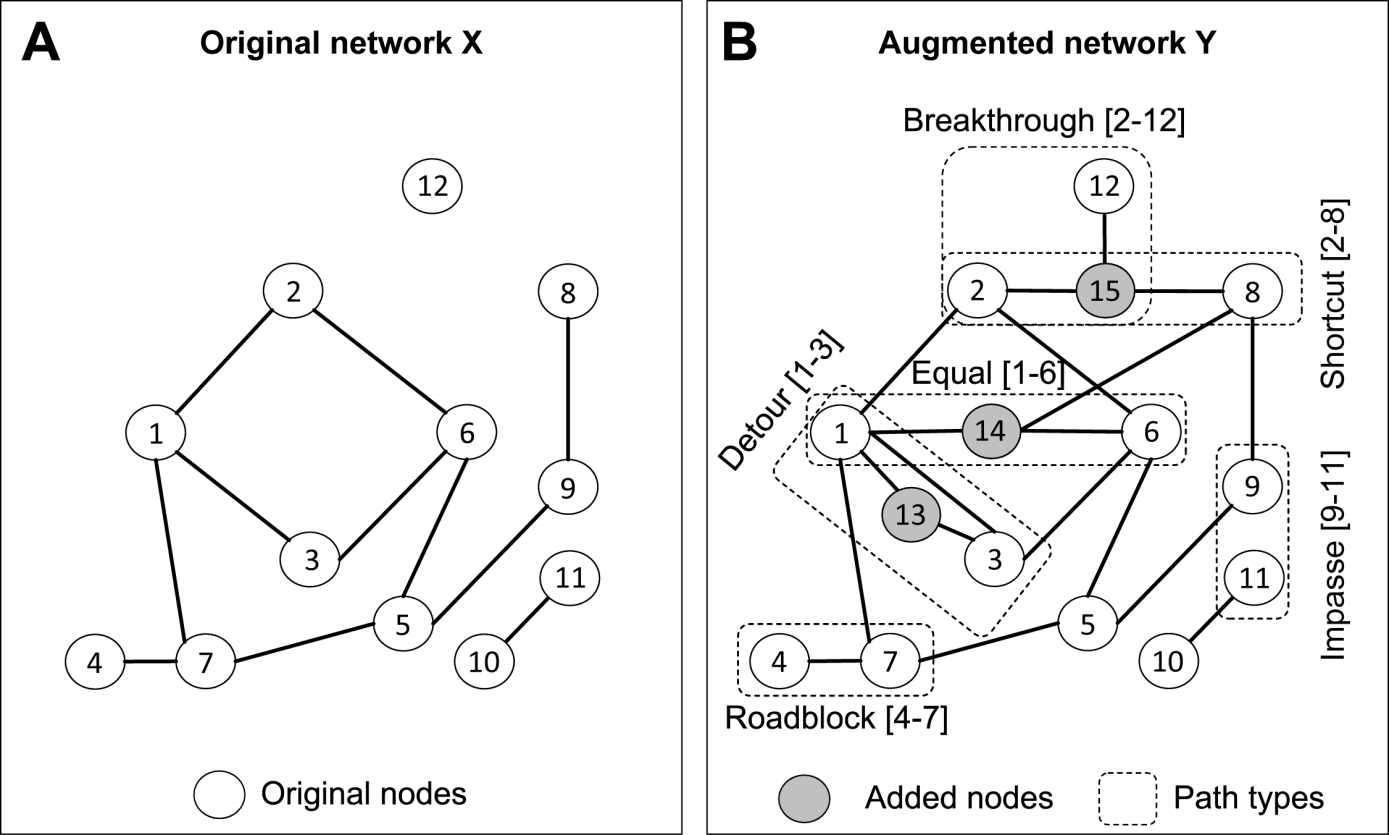
Examples of the BRIDES (Breakthrough, Roadblock, Impasse, Detour, Equal and Shortcut) paths in evolving networks. Panel (A) presents an original network *X* with 12 nodes. Panel (B) presents an augmented network *Y* with 15 nodes (including 12 original and 3 added nodes, which are colored in *grey*). Six different types of paths are shown in the augmented network *Y*.

**Fig 2:**
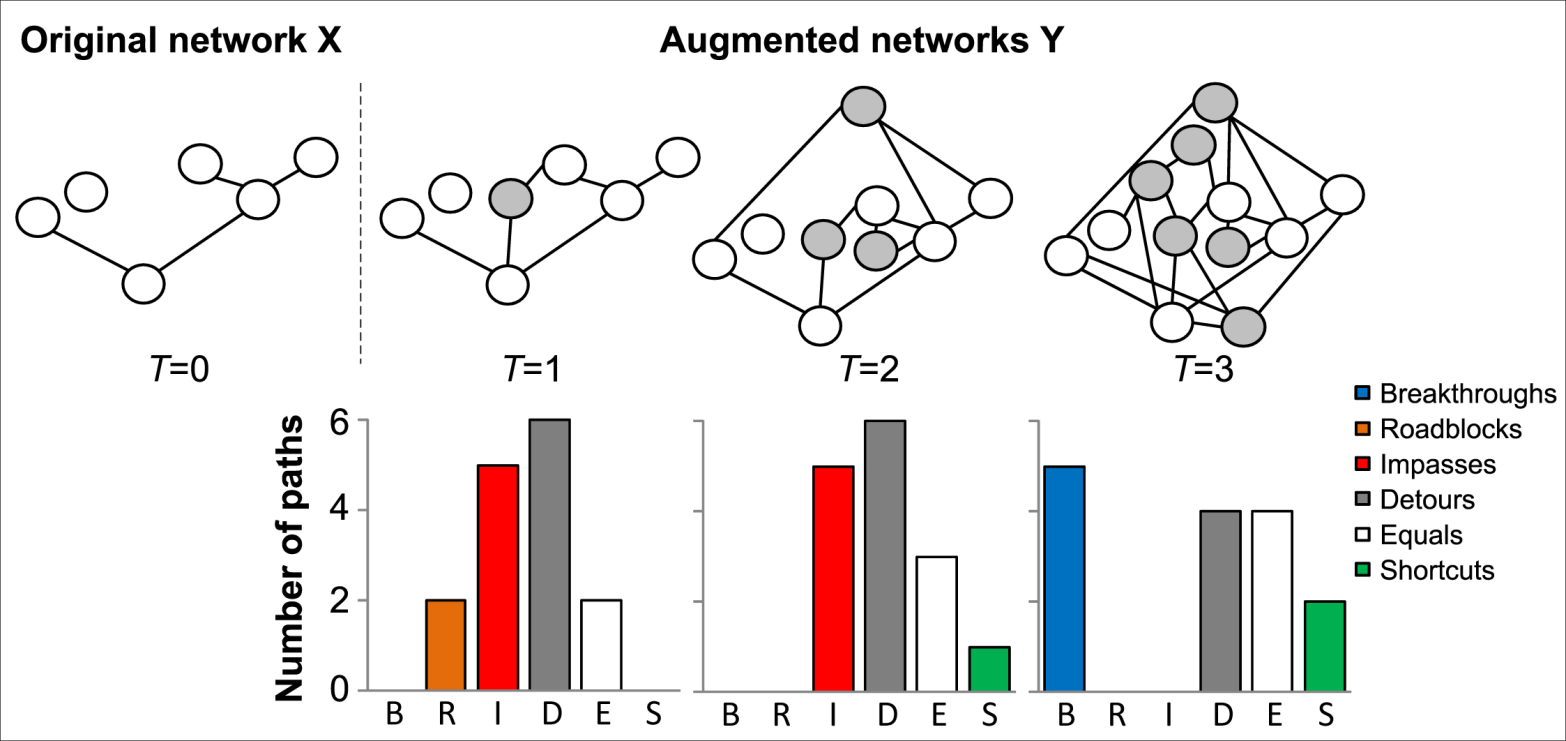
Computation of the BRIDES statistics in evolving networks. Addition of the new nodes (colored in *grey*) and edges to an evolving network changes the distribution of different types of network pathways as time (*T*) progresses. The letters B, R, I, D, E and S at the bottom of the chart stand respectively for Breakthroughs, Roadblocks, Impasses, Detours, Equal paths and Shortcuts.

The BRIDES algorithm takes as input two networks: (1) network *X* with an original set of nodes *N*_*X*_ and edges *E*_*X*_ (Fig 1A) and (2) network *Y* with an augmented set of nodes *N*_*Y*_ and edges *E*_*Y*_ (Fig 1B). All nodes of network *X* should also be present in network *Y* (i.e. *N*_*X*_ ⊂ *N*_*Y*_). However, it is not required that *E*_*X*_ ⊂ *E*_*Y*._. We first compute the shortest paths between the pairs of nodes in *X*, and then reassess their length in *Y*, by forcing these paths to include at least one added node (i.e. a node present in *Y*, but not in *X*). Our heuristic relies on a repeated application of Dijkstra's algorithm [21] to evaluate the impact of added nodes to the length of the shortest paths between the original nodes (see Algorithm 1).

Now we can define six distinct types of paths, related to the existence of added nodes in *Y*, which can be used to characterize complex relationships in evolving networks (Fig 1):

**B**reakthrough: a path that is impossible in network *X* but is possible in network *Y* (e.g. path between nodes 2 and 12, passing by added node 15 in Fig 1B);

**R**oadblock: a path that is possible in network *X* but is impossible in network *Y* (e.g. a simple path between nodes 4 and 7 that passes by an added node in *Y* is impossible, see Fig 1B);

**I**mpasse: a path that is impossible in both networks, *X* and *Y* (e.g. there are no possible paths between nodes 9 and 11 in Fig 1A and 1B);

**D**etour: a path that is shorter in network *X* than in network *Y* (e.g. path between nodes 1 and 3 in Fig 1A and 1B);

**E**qual: a path that has the same length in networks *X* and *Y* (e.g. path between nodes 1 and 6, assuming that all edge lengths in *X* and *Y* are equal, Fig 1A and 1B);

**S**hortcut: a path that is longer in network *X* than in network *Y* (e.g. path between nodes 2 and 8 in Fig 1A and 1B).

Fig 2 provides an example of computation of the BRIDES statistics in evolving networks. We can see that the addition of new nodes and edges to an evolving network can substantially change the distribution of the six types of paths defined in our study. The four heuristic strategies tested in our simulations, called BRIDES (the original strategy), BRIDES_Y, BRIDES_YC and BRIDES_EC, are presented in details in Algorithm 1 below:

**Algorithm 1**

Given an original undirected network X = (E_X_, N_X_) and its augmented undirected network Y = (E_Y_, N_Y_), i.e. network such that N_X_ ⊂ N_Y_, this algorithm calculates the number of Breakthroughs, Roadblocks, Impasses, Detours, Equal paths and Shortcuts (BRIDES) to characterize the evolution of X into Y.

BRIDES

**Step 1.** Compute the length of the shortest path between all pairs of nodes in network *X*. Find at most *MaxPathNumber* of simple shortest paths between pairs of original nodes (*i*,*j*), (i.e. nodes such that *i* ∈ *N*_*X*_ and *j* ∈ *N*_*X*_) in network *Y*, using Dijkstra's algorithm. Store in the list *P*_*ij*_ the set of simple shortest paths corresponding to a pair of original nodes (*i*,*j*) in network *Y*.

**Step 2.** For all pairs of original nodes (*i*,*j*), create a list *L*_*ij*_ of added nodes *k* (*k* ∈ *N*_*Y*_, *k* ∉ *N*_*X*_) ordered with respect to the closeness of *i* and *j* to *k*. Calculate the distances *d*(*i*,*k*) and *d*(*j*,*k*) in *Y* using Dijkstra and store at most *MaxPathNumber* of simple shortest paths in *P*_*ik*_ and *P*_*jk*,_ respectively. Order the list of added nodes *L*_*ij*_ according to either the minimum of Max(*d*(*i*,*k*), *d*(*j*,*k*)) (Strategy 1 that provided better overall results in our simulations; the results of this strategy will be presented in the next section) or the minimum of (*d*(*i*,*k*) + *d*(*j*,*k*)) (Strategy 2). In order to speed up the algorithm, we can reduce the size of *L*_*ij*_ by using the input parameters: *MaxDistance* (the maximum allowed distance from *i* or from *j* to *k*) and/or *MaxNode* (the maximum number of added nodes, *k*, in this list).

Set the first pair of original nodes (*i*,*j*) as the *current pair of nodes*.

**Step 3.** Do, for the current pair of original nodes (*i*,*j*):

If there exists a simple path in *P*_*ij*_ that includes at least one added node, update the BRIDES statistics (see Table 1) with the results obtained for the current pair of nodes (*i*,*j*), set the next pair (*i*,*j*) as the current pair of original nodes and go to the beginning of Step 3; otherwise, go to Step 4.

**Step 4.** At this point, we have determined that the current pair of original nodes (*i*,*j*) is not associated with a Breakthrough, Impasse or Shortcut, and we can now determine whether it should be associated with a Detour, Equal path or Roadblock.

Do, for each node *k* of the ordered list *L*_*ij*_, starting from the first element of *L*_*ij*_:

Step 4.1. If the *concatenation* of paths [*i*,*k*] from *P*_*ik*_ and [*j*,*k*] from *P*_*jk*_ is a simple path, set *d*(*i*,*j*) = *d*(*i*,*k*) + *d*(*j*,*k*) and update the BRIDES statistics (see Table 1) with the results obtained for the pair of nodes (*i*,*j*), set the next pair (*i*,*j*) as the current pair of original nodes and go to Step 3; otherwise, go to Step 4.2.

Step 4.2. Since there are repeating nodes, except *k*, on the paths [*i*,*k*] and [*j*,*k*], temporarily remove them from network *Y* and recalculate: (1) the shortest path from *j* to *k* in the reduced network *Y* using Dijkstra, storing the result in *P*_*jk*_', and (2) the shortest path from *i* to *k* in the reduced network *Y* using Dijkstra, storing the result in *P*_*ik*_'. Repeat Step 4.1 with the shortest of concatenations of two paths stored: (1) in *P*_*ik*_ and *P*_*jk*_' and (2) in *P*_*jk*_ and *P*_*ik*_'; if for both *P*_*jk*_' and *P*_*ik*_' does not return a simple shortest path because no such path exists in the reduced network *Y*, consider the next node *k* of *L*_*ij*_ in Step 4.1 or go to Step 5 if all element of *L*_*ij*_ have been already examined.

**Step 5.** Classify the path associated with the current pair of nodes (*i*,*j*) as a Roadblock.

**Step 6.** If all the pairs (*i*,*j*) have been already examined, print the BRIDES statistics; otherwise, set the next pair (*i*,*j*) as the current pair of original nodes and go to Step 3.

**Heuristic BRIDES_Y** (*BRIDES using Yen's algorithm*)

Replace Steps 3 and 4 above by the following steps:

**Step 3'**. Do, for the current pair of original nodes (*i*,*j*):

Compute the ordered list *PY*_*ij*_ of *MaxPathNumber* shortest paths between *i* and *j* using Yen's *k*-shortest path algorithm [22].

**Step 4'**. Do, for each path *p* of the ordered list *PY*_*ij*,_:

If *p* contains at least one added node, update the BRIDES statistics (see Table 1) with the results obtained for the current pair of original nodes (*i*,*j*), set the next pair (*i*,*j*) as the current pair of original nodes and go to Step 3.

**Heuristic BRIDES_YC** (*BRIDES using Yen's algorithm and Concatenation of paths*)

Replace Steps 2 and 4 above by the following steps:

**Step 2'.** For all pairs of original nodes (*i*,*j*), create a list *L*_*ij*_ of added nodes *k* (*k* ∈ *N*_*Y*_, *k* ∉ *N*_*X*_) ordered with respect to the closeness of *i* and *j* to *k*. Calculate the distances *d*(*i*,*k*) and *d*(*j*,*k*) in *Y* using Dijkstra. Order the list of added nodes *L*_*ij*_ according to the minimum of Max(*d*(*i*,*k*), *d*(*j*,*k*)) (Strategy 1) or the minimum of (*d*(*i*,*k*) + *d*(*j*,*k*)) (Strategy 2). Using Yen's algorithm compute and store at most *MaxPathNumber* of paths in *P*_*ik*_ and *P*_*jk*_, respectively. In order to speed up the algorithm, we can reduce the size of *L*_*ij*_ by using the input parameters: *MaxDistance* and/or *MaxNode*.

Set the first pair of original nodes (*i*,*j*) as the current pair of nodes.

**Step 4'.** Do, for each node *k* of the ordered list *L*_*ij*_, starting from the first element of *L*_*ij*_:

Step 4.1. If the *concatenation* of paths [*i*,*k*] from *P*_*ik*_ and [*j*,*k*] from *P*_*jk*_ is a simple path, set *d*(*i*,*j*) = *d*(*i*,*k*) + *d*(*j*,*k*) and update the BRIDES statistics (see Table 1) with the results obtained for the pair of nodes (*i*,*j*), set the next pair (*i*,*j*) as the current pair of original nodes and go to Step 3.

Go to Step 5 if all element of *L*_*ij*_ have been already examined.

**BRIDES_EC** (*BRIDES algorithm based on an Exhaustive Concatenation approach*)

The difference with the original BRIDES algorithm is in Step 4, where we examine all the nodes *k* of the ordered list *L*_*ij*_, even though a simple path has been found in Step 4.1.

**Table 1:** Possible BRIDES outcomes depending on the path type identified by the algorithm.

*Simple path between i to j in X*	*Simple path between i to j in Y*	*BRIDES statistic*
Impossible	Possible	Breakthrough
Possible	Impossible	Roadblock
Impossible	Impossible	Impasse
Shorter distance	Longer distance	Detour
Equal distance	Equal distance	Equal
Longer distance	Shorter distance	Shortcut

The default values of the parameters *MaxPathNumber*, *MaxDistance* and *MaxNode* in our program are all equal to 100. These default values were also used in our simulation study (see the next section). It is worth noting that in unweighted graphs, *MaxDistance* represents the upper bound of the number of edges on the path between an original and an added node. Obviously, in weighted graphs, this parameter should be specified by the user. The time complexity of Steps 1 and 2 of our original BRIDES algorithm is *O*(|*N*_*X*_| × *MaxPathNumber* × (|*E*_*Y*_| + |*N*_*Y*_|log(|*N*_*Y*_|)), using asymptotically the fastest known single-source shortest-path version of Dijkstra's algorithm, where |*N*_*X*_| and |*N*_*Y*_| are the numbers of nodes in networks *X* and *Y*, respectively, and |*E*_*Y*_| is the number of edges in network *Y*. The time complexity of Step 4 is *O*(|*N*_*X*_|^2^ × *MaxNodes* × (|*E*_*Y*_| + |*N*_*Y*_|log(|*N*_*Y*_|)), in the worst case. However, in practice, the runtime of this step is much lower because we rarely execute the internal loop of Step 4 all the *MaxNodes* times. This leads to the total time complexity of BRIDES equal to *O*(|*N*_*X*_| × (*MaxPathNumber* + |*N*_*X*_| × *MaxNodes*) × (|*E*_*Y*_| + |*N*_*Y*_|log(|*N*_*Y*_|)). The presented BRIDES algorithm can be applied to analyze undirected graphs with non-negative edge lengths. When negative edge lengths exist in either network *X* or network *Y*, the improved version of Bellman–Ford’s algorithm [23] could be applied instead of Dijkstra.

We created an R function implementing the BRIDES algorithm using the graph manipulation tools available in the *igraph* package [24]. The R version of BRIDES can be applied for the analysis of small networks (<1,000 nodes). For larger networks (>1,000 nodes), we recommend using the C++ version of our program, which includes all the four heuristic algorithms, BRIDES, BRIDES_Y, BRIDES_YC and BRIDES_EC, discussed in this paper. Moreover, a parallel OpenMP [25] version of the C++ program is also available (see: https://github.com/etiennelord/BRIDES).

## Results

### Simulation study

To test the BRIDES algorithm, we carried out a simulation study using three popular network models implemented in the *igraph* package (version 1.0.0) [24]. Precisely, the Erdős–Rényi [26], Barabási–Albert [27] and Watts–Strogatz [28] random graph generation models were considered. The Barabási–Albert model is a well-known approach for generating scale-free (or power-law) networks, while the Erdős–Rényi and Watts–Strogatz models are among the most popular generation models for random graphs that do not exhibit a scale-free degree distribution.

**Fig 3:**
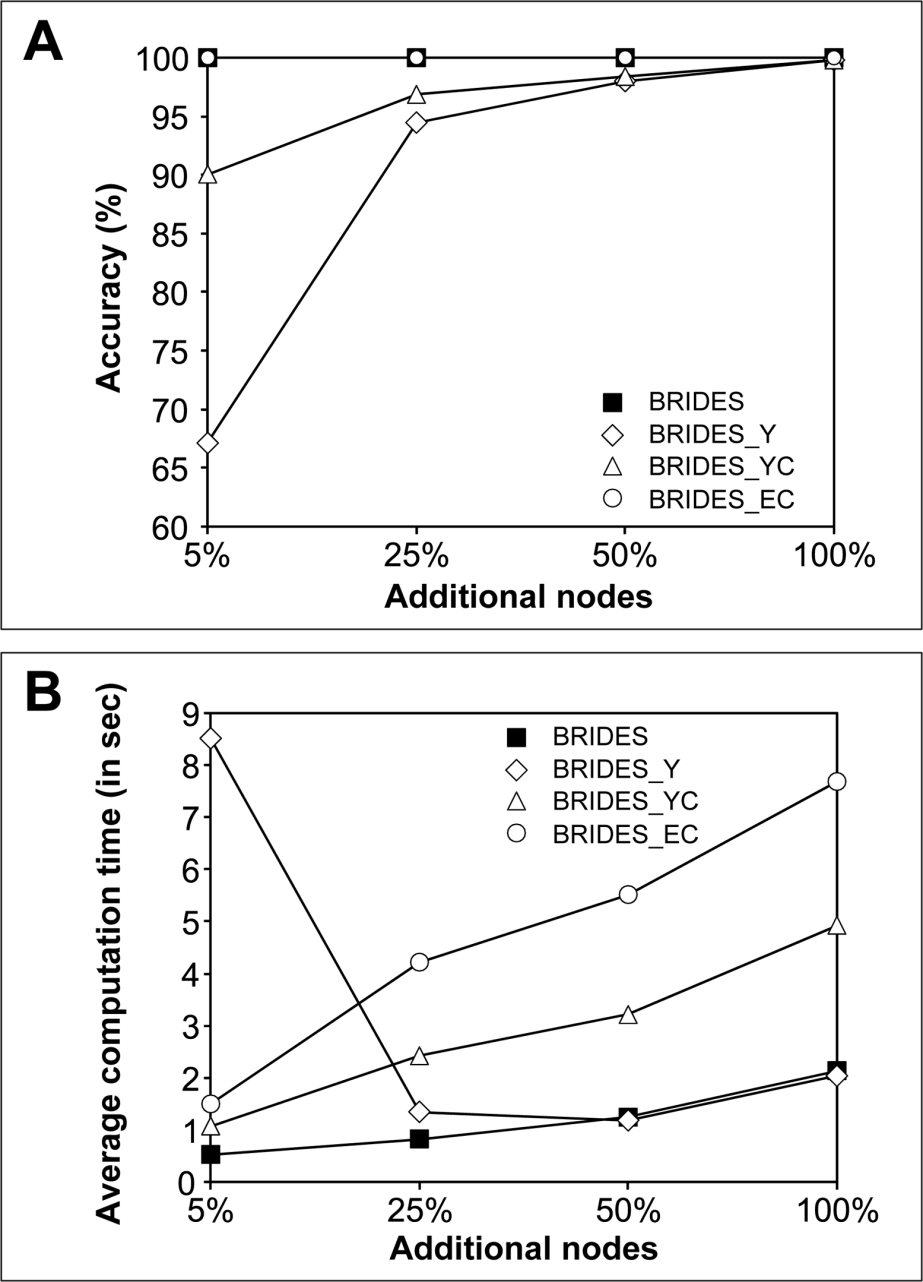
**The average accuracy (A) and computational time (B) obtained for the four heuristic strategies, BRIDES, BRIDES_Y, BRIDES_YC and BRIDES_EC, presented in our study.** The simulations were carried out with original networks *X* containing 100 nodes and generated using the Erdős–Rényi, Barabási–Albert and Watts–Strogatz random graph generation models. The augmented networks *Y* contained 5, 25, 50 or 100 added nodes and all original nodes of *X*. 1000 graphs were generated for each parameter configuration.

Using each of these three models, we generated 1000 random original networks *X* with 100 nodes, and then added to them 5, 25, 50 or 100 additional nodes to create augmented networks *Y*. The simulations were performed using the C++ version of our program executed on a PC computer equipped with an Intel i7-3770 CPU (3.40GHz) and 8Gb of RAM. The four competing heuristic strategies, BRIDES, BRIDES_Y, BRIDES_YC and BRIDES_EC, presented in Algorithm 1 were tested in our simulation study. The accuracy of the competing approaches (see Fig 3A) was calculated as the percentage of correctly labeled path types (i.e. the percentage of true positive Breakthroughs, Roadblocks, Impasses, Detours, Equal paths and Shortcuts) provided by each heuristic. The identification of the correct (i.e. reference) path types was carried out using a brute force procedure based on a depth-first search (DSF) algorithm. Along with the average accuracy, calculated over all generated graphs, we also measured the average runtime (in seconds; see Fig 3B and S1 Table) of each of the four heuristics under comparison.

The results of our simulations suggest that the original BRIDES strategy along with the exhaustive concatenation procedure, BRIDES_EC, were able to provide the correct classification of paths, regardless of the number (i.e. also percentage, in this case) of added nodes (Fig 3A). The two heuristics based on Yen’s *k*-shortest path algorithm (i.e. BRIDES_Y and BRIDES_YC), returned the correct classification of paths in 67% and 90% of cases, respectively, when the number of added nodes was 5. However, the results obtained with BRIDES_Y and BRIDES_YC improved with an increase of the number of added nodes, reaching the accuracy level of 100% for 100 added nodes.

The original BRIDES algorithm was the fastest among the four compared heuristics regardless the number of added nodes (Fig 3B). The worst performance, in terms of running time, was shown by BRIDES_Y, for small numbers of added nodes. This can be explained as follows. On one hand, we restrict our search in Yen’s algorithm to only 100 shortest paths. On the other hand, when the proportion of added nodes in *Y* is very small, all of them can be located far away from both source and destination nodes given as input to Yen’s algorithm. Thus, these rare added nodes can never be included in the set of 100 shortest paths returned by BRIDES_Y. This leads to an important decrease in accuracy (Fig 3A) and increase in computational time (Fig 3B). The heuristic BRIDES_YC, which uses both Yen's algorithm and concatenation of paths, performs better in this case since the concatenated paths are guaranteed to contain at least one added node.

It is worth noting that the length of a Detour path identified by the BRIDES algorithm can be longer than the length of the shortest possible Detour existing in the network, but in this work, we are only interested in estimating the distribution of path types, and not in assessing the exact path lengths.

### Application of BRIDES to real biological data

**Fig 4:**
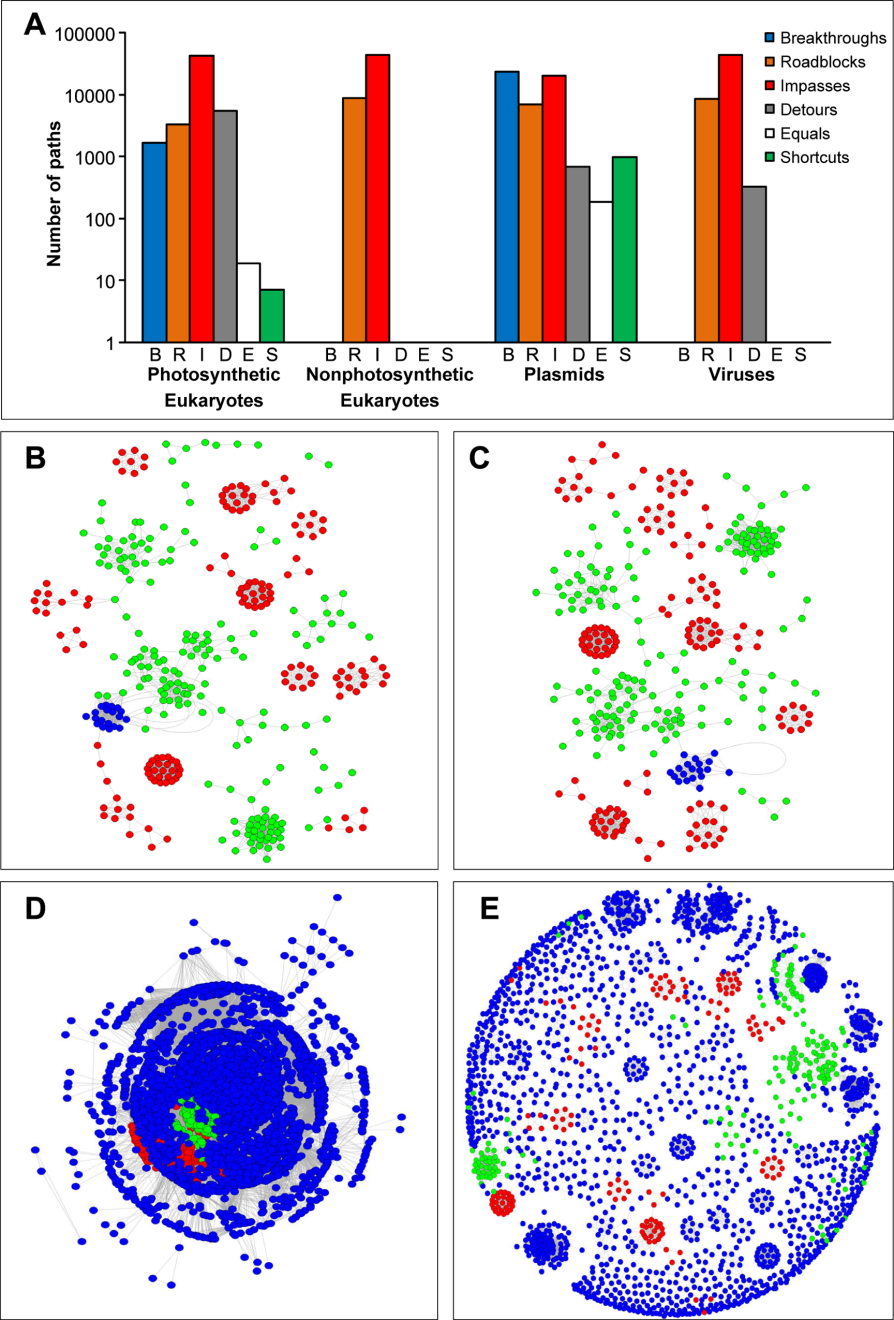
BRIDES statistics for real genome similarity networks at 90% similarity threshold. The BRIDES statistics (A) computed for the original network *X* comprising archaea (in *red*) and bacteria (in *green*), 326 species in total, and the four augmented similarity networks *Y* (the added nodes are in *blue*), including: (B) photosynthetic eukaryotes, (C) nonphotosynthetic eukaryotes, (D) plasmids and (E) viruses.

To evaluate the performance of the BRIDES algorithm and calculate the related statistics (Fig 4A) for real networks, we generated four genome similarity networks using a set of 2,094,593 nucleotide sequences of archaea, bacteria, photosynthetic and nonphotosynthetic eukaryotes, plasmids and viruses. Similarity between the nucleotide sequences was determined using *BLAST* [29] with a minimum *e*-value of 10*e*-5. Individual genomes were connected in both original and augmented networks if at least one of their genes shared a homologous sequence (>70% cover, with the 90% similarity threshold). A total of 326 prokaryotes were selected to form the original network *X*, and then complemented with either photosynthetic eukaryotes (Fig 4B), or nonphotosynthetic eukaryotes (Fig 4C), or plasmids (Fig 4D), or viruses (Fig 4E) in the augmented network *Y*.

The BRIDES statistics provided by our algorithm for the four genome similarity networks exhibited different distribution profiles (Fig 4A). Even when the genome networks displayed similar clustering coefficients and comparable average path lengths (Table 2), the BRIDES statistics could be quite different (Fig 4A). For example, the addition of photosynthetic eukaryotes resulted in a large number of Detours, Shortcuts and Equal paths (Fig 4B), whereas the addition of nonphotosynthetic eukaryotes resulted in the complete disappearance of such path types (Fig 4C). This difference can be explained by the fact that nuclear genomes of photosynthetic eukaryotes host gene families from cyanobacterial origin, as a result of gene transfer from their chloroplastic endosymbionts, which are typically absent in nonphotosynthetic eukaryotes. Moreover, the addition of 3552 plasmids (Fig 4D) to the original network *X* led to a similar BRIDES profile as in the case of photosynthetic eukaryotes, while favouring the emergence of Shortcuts. On the contrary, the addition of viruses (Fig 4E) did not introduce any Shortcuts into the augmented similarity network, but led to the increase of the numbers of Roadblocks. The latter result is consistent with findings of Halary and colleagues [8], who identified plasmids as central genetic carriers across prokaryotic genomes, whereas viruses were found to have restricted host ranges for infecting distantly related taxa.

**Table 2:** General network and BRIDES statistics for the four real genome similarity networks presented in Fig 4.

	*General network statistics*					*BRIDES statistics*^a^					
Added species	Total of nodes	Total of edges	Average degree	Average path length	Clustering coefficient	B	R	I	D	E	S
Eukaryotes photosynt.	345	2,014	11.68	5.97	0.867	1,652	3,329	42,418	5,550	19	7
Eukaryotes nonphotosynt.	345	1,845	10.70	5.29	0.865	0	8,905	44,070	0	0	0
Plasmids	3,848	187,848	97.63	2.90	0.559	23,618	7,057	20,452	689	185	974
Viruses	1,984	12,054	12.15	5.23	0.801	0	8,577	44,070	328	0	0

^a^ Computation were carried out using the following input parameters: *MaxDistance* = 100, *MaxNode* = 100 and *MaxPathNumber* = 100.

## Conclusion

In this paper, we introduced a new fast algorithm and associated software for characterizing different types of paths existing in evolving similarity networks. In particular, our algorithm calculates the number of Breakthroughs, Roadblocks, Impasses, Detours, Equal paths and Shortcuts (BRIDES), which can be present in these networks. Our program, implemented in the C++ and R programming languages, includes four heuristic algorithms for calculating the BRIDES statistics discussed and compared in our study (see Algorithm 1). These statistics can be viewed as a new tool for the characterization and comparison of evolving genome and gene similarity networks, transcriptional networks [30] or interactome networks [31]. The analysis and comparison of evolving networks can be carried out for different network stringency thresholds. Dijkstra’s algorithm used in our method makes it suitable for the analysis of both weighted and unweighted types of networks. Note that our BRIDES heuristic presented here in the case of undirected networks can be easily adapted to the case of directed networks. In the future, it would be interesting to see whether the Uniform Cost Search [32] or A* [33,34] algorithms can be used as an alternative of Dijkstra in order to accelerate the computation of the BRIDES statistics within our method.

## Dataset and Source Files

The R and C++ source codes are available from the Github repository (https://github.com/etiennelord/BRIDES/) with a GPL version 3 license. The sample networks (Figs 1 and 2) are located in either the R or C++ source directories. The source code for the simulations (Fig 3) is located in the Simulation directory of the github repository. The genome similarity networks (Fig 4, Table 2) are available in the GenomeNetwork directory.

## Supporting Information

S1 Table Average computational time in seconds (s) obtained for the four heuristics and for different network models. The original network *X* contained 100 nodes and the augmented networks *Y* contained 5, 25, 50 or 100 added nodes. For each model, 1000 networks were created, and 100 path were randomly selected for evaluation. The reported values are average time (in seconds) for the evaluation of each path.
(PDF)
