# Supplementary material for: BRIDES: A New Fast Algorithm and Software for Characterizing Evolving Similarity Networks Using Breakthroughs, Roadblocks, Impasses, Detours, Equals and Shortcuts
Source: PLoS One. 2016 Aug 31;11(8):e0161474. doi: 10.1371/journal.pone.0161474 (PMC5007014; doi:10.1371/journal.pone.0161474)
Supplement: S1 Table — The original network X contained 100 nodes and the augmented networks Y contained 5, 25, 50 or 100 added nodes. For each model, 1000 networks were created, and 100 path were randomly selected for evaluation. The reported values are average time (in seconds) for the evaluation of each path. (PDF) [file pone.0161474.s001.pdf]

S1 Table. Average computational time in seconds (s) obtained for the four heuristics and for different network models. The original network *X* contained 100 nodes and the augmented networks *Y* contained 5, 25, 50 or 100 added nodes. For each model, 1000 networks were created, and 100 path were randomly selected for evaluation. The reported values are average time (in seconds) for the evaluation of each path.

Heuristics	*Network model*	*Added nodes*			
		**5**	**25**	**50**	**100**
**BRIDES**	Erdős–Rényi model	0.081	0.132	0.175	0.270
	Barabási–Albert model	0.082	0.146	0.203	0.296
	Watts–Strogatz model	0.061	0.067	0.067	0.076
**BRIDES_Y**	Erdős–Rényi model	1.535	0.134	0.077	0.072
	Barabási–Albert model	1.725	0.244	0.129	0.085
	Watts–Strogatz model	0.112	0.050	0.047	0.049
**BRIDES_YC**	Erdős–Rényi model	9.943	7.211	4.711	4.491
	Barabási–Albert model	8.343	7.093	6.446	5.975
	Watts–Strogatz model	2.198	3.260	2.897	2.229
**BRIDES_EC**	Erdős–Rényi model	0.153	0.386	0.622	1.060
	Barabási–Albert model	0.212	0.701	1.236	2.263
	Watts–Strogatz model	0.066	0.080	0.086	0.108
